# Uncovering the Carbon Emission Intensity and Reduction Potentials of the Metro Operation Phase: A Case Study in Shenzhen Megacity

**DOI:** 10.3390/ijerph20010206

**Published:** 2022-12-23

**Authors:** Kunyang Chen, Guobin Zhang, Huanyu Wu, Ruichang Mao, Xiangsheng Chen

**Affiliations:** 1 Key Laboratory for Resilient Infrastructures of Coastal Cities, Ministry of Education, Underground Polis Academy, College of Civil & Transportation Engineering, Shenzhen University, Shenzhen 518061, China; 2 Shenzhen Key Laboratory of Green, Efficient and Intelligent Construction of Underground Metro Station, Shenzhen University, Shenzhen 518060, China; 3 State Environmental Protection Key Laboratory of Mineral Metallurgical Resources Utilization and Pollution Control, Wuhan University of Science and Technology, Wuhan 430081, China; 4 School of Civil Engineering, Tsinghua University, Beijing 100084, China

**Keywords:** metro, operation phase, carbon emission intensity, reduction potential, scenario analysis

## Abstract

The huge energy consumption of metro operations has become a significant challenge faced by the urban public transportation sector to achieve low-carbon development. Using Shenzhen as an example, this study has made efforts to quantify the metro’s energy consumption and carbon emission intensity during the operation phase by using the Life Cycle Assessment approach. Furthermore, this study evaluates the actions that can be taken to reduce energy consumption and emissions. A comparative analysis between metros and other public transportation modes has also been conducted. The results show that the annual carbon emissions from the metro’s operation phase in Shenzhen city increased from 63,000 t CO_2_e in 2005 to 1.3 Mt CO_2_e in 2021, and the historically accumulated carbon emissions are 9.5 Mt CO_2_e. The unit operating mileage, the unit station area, and the per capita carbon emission intensity were 2.1 kg CO_2_e/km, 132.5 kg CO_2_e/m^2^, and 0.6 kg CO_2_e per capita (13th Five-Year Plan Period), respectively. By continually promoting the low-carbon operation of the subway, the cumulative carbon savings could reach 0.1 Mt CO_2_e (2022–2035).

## 1. Introduction

Coping with global warming is a major focus in today’s world. In its 14th Five-Year Plan, China proposed the goal of “achieving peak carbon by 2030 and striving to achieve carbon neutrality by 2060”. The implementation of the “double carbon” goal is of great strategic significance for promoting a comprehensive green transformation of China’s economy and society and building a harmonious symbiotic development between humans and nature.

Transportation is a significant source of greenhouse gas emissions. According to the data released by the International Energy Agency (IEA), the energy consumption of the global transportation sector was 21% in 2018, and the carbon emissions it generated made up about 23% [1]. In terms of domestic transportation, the carbon emissions of China’s transportation system accounted for about 9% of the total carbon emissions of all industries in 2019 [2]. Both at home and abroad, the transportation sector has become the main source of China’s energy consumption, affecting urban air quality and increasing carbon emissions [3]. In order to cope with climate change and promote sustainable, low-carbon transformation in the field of transportation, China has attached great importance to the development of public transportation and stipulated that the passenger share of carbon emissions from public transportation in major cities should reach 30% [4].

**Figure 1:**
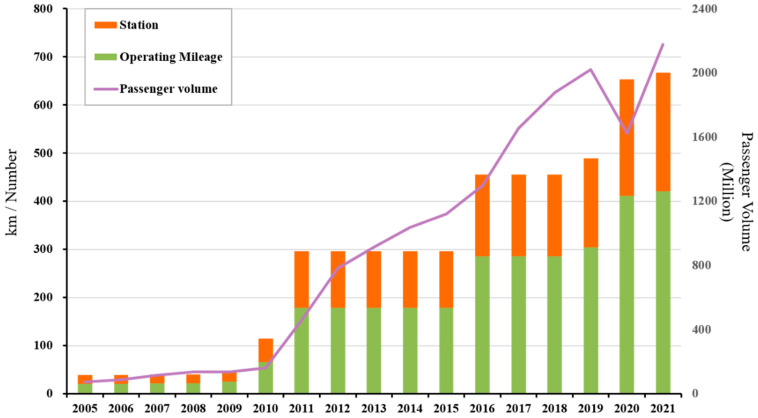
Mileage, number of stations, and passenger volume of Shenzhen Metro from 2005 to 2021.

As one of the rapidly developing cities in China, due to the rapid expansion of the transportation industry and the growth of the urban population (from 8.3 in 2005 to 17.6 million in 2020), Shenzhen is facing major environmental challenges that are similar to other megacities in China [5]. Specifically, the mileage of the metro system in Shenzhen has expanded from 20.9 km at the end of 2004 to 419 km in 2021. By the end of 2021, a total of 11 lines and 265 stations (excluding MTR line 4) had been completed and put into service. Passenger volume has also increased from 730,000 in 2005 to 2.18 billion in 2021, as shown in Figure 1. As a large-capacity and highly punctual public transportation mode, the metro system alleviates not only urban traffic congestion and improves the convenience of urban public travel, but also introduces significant challenges regarding the urban environment, energy security, and public health [6,7,8]. In the future, Shenzhen Metro will continue to promote new road network planning and construction [9]. Given the importance of this metro system, methods for reasonably quantifying and predicting its carbon emission, and exploring the potential for reducing emissions, are problems that urgently need to be solved.

So far, most studies have focused on the environmental impacts of public transportation systems [10,11] and have carried out resource and energy analyses and carbon emissions research on different transportation modes. Private automobiles are one of the most common modes of transportation in cities worldwide, including China. Liu et al. (2018) evaluated the impact of fuel cell vehicles on China’s road-transport greenhouse gas emissions [12]. The research results demonstrated that electric or hydrogen vehicles have the strongest potential for reducing emissions. One approach for reducing the environmental impact of passenger transportation is vehicle sharing, and in recent years, shared bicycles and cars have aroused interest in academic circles due to their environmentally protective performance [13,14]. Mao et al. (2021) pointed out that the production stage of shared bicycles accounts for 81.18% of the environmental impacts of their whole life cycle, and the use of aluminum accounts for about 55.43% of the environmental impact of the production stage, comprising the largest contribution [15]. Some studies have found that in terms of greenhouse gas emissions, shared cars may not be as environmentally friendly as expected [16]. Hence, to meet the growing transportation needs, urban public transportation in general, and metro systems in particular, are some of the most resource-saving schemes compared with other transportation systems. Pero et al. (2015) studied the greenhouse gas emissions and energy consumption of the whole life cycle of Rome’s metro system. The results showed that the energy consumption intensity in the use stage accounts for about 98.3% of the total electricity consumption in the vehicle cycle [17]. Lajunen and Lipman (2016) quantified the carbon emissions of buses powered by various forms of energy from the perspective of life cycle cost. From these studies, it has been found that a life-cycle assessment is suitable for quantifying the carbon emissions of public transportation systems [18].

Some studies have carried out research on the characteristics of carbon emissions in the field of transportation at different scales. Mao et al. (2021) describe a metro development model at the global scale, building a material inventory and determining the actual carbon emissions of 219 cities throughout the world that have metro systems [19]. At the national level [20], Duan et al. (2015) tried to quantify the environmental impacts of multiple transportation sectors, such as the railway, highway, waterway, and air transportation sectors [21]. Xu and Lin (2016) investigated the key drivers of carbon emissions in China’s transportation sector at the regional level; the results showed that the impact of urbanization on carbon emissions from the transportation sector trended downward from the western region to the eastern and central regions [22]. At the municipal level [23,24,25,26,27], Duan et al. (2017) used a “bottom-up” method to study the carbon emissions of Shenzhen’s transportation system; their results showed that the carbon emissions from the public transportation system accounted for 24% of the entire transportation sector [28]. Dong et al. (2018) studied the carbon emissions of Shenzhen’s public transportation system. They found that buses and metros accounted for 64% and 36%, respectively, of the total carbon emissions of this urban public transportation system, although the study ignored the impact of taxis [29]. None of the above studies, however, analyzed the carbon emissions or emission reduction potential of public transportation systems, especially metro systems, into the future—up to 2035.

As an important part of urban public transportation, the metro has been designated a cleaner commuting mode; its low carbon emissions have become one of its defining characteristics [30]. By comparing the carbon footprint of the commuter metro with those of other commuter modes, the ability of the metro to play a major role in reducing greenhouse gas emissions has been confirmed [31,32].

From the life-cycle perspective, operational activities are the main source of greenhouse gas emissions from metros. Li et al. (2018) pointed out that the carbon emissions in the metro construction stage accounts for less than 4.6%, the operational stage accounts for 92.1%, and the maintenance stage accounts for 3.4% [33]. Therefore, it is necessary to deeply explore the carbon emission characteristics and emission reduction potential of the metro system in the operational stage, whereas the existing relevant research has mainly focused on metro infrastructure construction [30,34]. It can also be seen that the existing metro-related research has mainly focused on specific projects or characteristic stages and has overlooked its ability to reduce carbon emissions. Moreover, few studies have measured or compared carbon emissions at the city level, attempted to predict future carbon emissions levels, or explored the metro’s emission reduction potential or development strategies.

Therefore, with the aim of meeting this unmet need, this study adopts the life-cycle assessment (LCA) method, selects the CO_2_ equivalent (CO_2_e) as the environmental impact assessment index, takes the metro operational stage as the research object, constructs a carbon emissions assessment method for the metro operational stage, and quantitatively analyzes energy consumption and the carbon emission intensity and level for metro operation. By comparing these indicators with those of other public transportation systems (buses and taxis) in Shenzhen and with the metro operations in other cities, the carbon emissions level of Shenzhen Metro operation is clarified. Finally, using the scenario analysis method, the carbon emissions of Shenzhen Metro in the operational period of 2022 to 2035 and the degree of achievable carbon emission reduction by implementing energy-saving and emission-reduction measures are predicted. The research results can provide theoretical methods and data references for researchers related to reducing the carbon emissions of the metro system, as well as a theoretical basis and data support for green and low-carbon transformation in the transportation field and a scientific formulation of the energy-saving management policies of the metro system by relevant government departments.

## 2. Methodology

**Figure 2:**
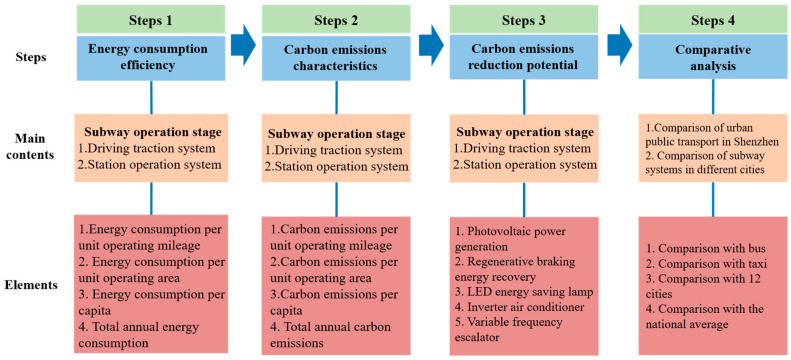
Environmental impacts assessment framework.

LCA is a quantitative analysis method of evaluating the potential comprehensive environmental impact of a product (or service or activity) from raw material mining to final disposal [35]. This method can be used to evaluate resource and energy consumption and the environmental impacts and benefits of the management process in the field of transportation. Carrying out LCA research includes four stages: (1) the determination of objectives and scope; (2) inventory analysis; (3) impact assessment; and (4) the interpretation of the results [36]. However, because ISO standards are defined in a common language, it is difficult to evaluate whether a particular LCA has been carried out step by step according to the standard [37]. This study, therefore, references some other LCA studies on metros and their environmental impacts [21,29,38]. Based on life-cycle theory, this study developed a model for evaluating the environmental impact of a metro’s operational stage. The research framework is shown in Figure 2.

### 2.1. Scope and System Boundary

**Figure 3:**
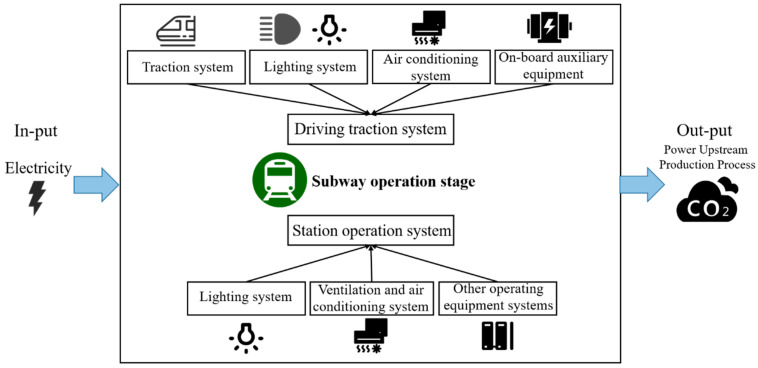
System boundary in metro operational stage.

Following the principles of ISO 14040/44 [39,40], this study selected CO_2_ equivalent (CO_2_e) as the environmental impact assessment index in order to assess the environmental impacts of metros in the operational stage from the perspective of the whole life cycle. The system boundary of the metro operational stage includes the driving traction system and the station operation system, as shown in Figure 3. In the operational stage of a metro, electricity consumption is the main factor affecting emissions, including traction consumption and the use of on-board auxiliary equipment (ventilation and air conditioning system, broadcasting system, information system, etc.). As for station operation, it mainly includes power, ventilation, air conditioning, and lighting systems but does not include the energy consumption of commercial blocks. Driving traction includes traction consumption and the use of auxiliary equipment (ventilation and air conditioning system, broadcasting system, information system, etc.); station operation mainly includes ventilation and air conditioning and lighting systems, but does not include energy consumption in commercial blocks. Unlike diesel and gasoline, electricity, as it is used here, is considered green energy and does not directly generate greenhouse gases. Therefore, to quantify the environmental impacts of the metro operational stage, it is necessary to calculate the carbon emissions generated by the upstream production stage of the electricity consumed by the various equipment and systems during the metro’s operational stage. In addition, the time boundary of this study is 2005–2021, as the Shenzhen Metro was officially opened on 28 December 2004.

In LCA research, environmental impact assessment needs to be carried out in terms of functional units, which can quantify the selected products and determine their functional characteristics according to the ISO 14040/44 series of standards. During the operation of the metro, the energy consumption and carbon emissions of the driving traction and station operation systems are quite different. In order to compare the metro operations of different cities and cases and make the results applicable to future research, the driving traction and the station operation during the metro operational stage are expressed as unit operating mileage (1 km), unit operating area (1 m^2^), and unit passenger capacity (per capita).

### 2.2. Carbon Emissions Calculation Method

The carbon emissions in the metro operational stage are the sum of the carbon emissions generated by the electricity consumption of the equipment and systems in the driving traction system and of the station operation system, as shown in Equation (1).

(1) $$CE_{ST\left(i\right)}=U_{t\left(i\right)}\times E_{k,e}+U_{s\left(i\right)}\times E_{k,e}$$

where $CE_{ST}$ is the carbon emissions of the metro system in the first year of operation (CO_2_e); $U_{t\left(i\right)}$ and $U_{s\left(i\right)}$ represent the electricity consumption of the metro operating traction system and the station operating system in the ith year, respectively (kWh); and $E_{k,e}$ is the carbon emission factor of electricity for the year (kg CO_2_e/kWh).

### 2.3. Data Inventory

This study selected Shenzhen Metro as a case study. The data types and source details of the operational stage are shown in Table S1. The electricity consumption was obtained through a field investigation of the Shenzhen Metro Corporation. The electricity consumption of the driving traction system came from the Monthly Statistical Report of the Shenzhen Metro Operation Energy Consumption, and the electricity consumption of the station operation system came from the Statistical Account of Shenzhen Metro Operation Energy Consumption (see Figure S1). Electricity is the primary energy source consumed during the operation of the metro. Taking into account the regional characteristics, the carbon emission factor of electricity refers to the baseline emission factor of China Southern Power Grid [41] (see Table S3). The length of the metro lines in operation, the metro operating mileage, and the number of stations and their scale are all from the annual report and survey of the Shenzhen Metro and are summarized here (Figure 1, Figures S2 and S3). The passenger volume of Shenzhen Metro from 2007 to 2009 can be obtained from the Annual Report of the Shenzhen Metro Group Co., Ltd., and the monthly passenger volume of Shenzhen Metro from 2009 to 2021 can be obtained from the Monthly Statistical Report of Transportation Production Indicators issued by the Transport Commission of Shenzhen Municipality. The annual passenger volume results can be obtained by addition [42] (see Table S2). This study also performed a statistical analysis of the construction in progress and the planned construction for the Shenzhen Metro [9,43] (see Figure S5) in order to calculate the consumption intensity of resources and energy and create a data basis for analyzing the emission reduction potential of the metro in the future. To compensate for unavailable statistics and data from some of the sources, the quality of the data and uncertainty had to be evaluated [21]. Uniform distribution is generally used in such cases. We assumed that the coefficient of variance (COV) is 10%, especially for carbon emission factors. The pedigree matrix used for the uncertainty estimation was used to quantify this COV value.

### 2.4. Scenario Setting

In 2019, about 68.1% of China’s electricity came from fossil-fuel energy generation [44]. Electricity consumption during the operation of metros will, therefore, generate significant carbon emissions in the upstream production stage. In recent years, the Shenzhen Metro has taken the lead in adopting technologies such as photovoltaic electricity generation systems, inverter air conditioners, inverter escalators, and regenerative braking energy recovery. Therefore, based on the results of the sensitivity analysis (see Figure S4) for the current energy-saving and emission-reduction measures and future planning policies, this study set four scenarios with 2021 as the reference year: the baseline scenario, Scenario I (Conservative), Scenario II (Moderate), and Scenario III (Optimistic) (see Table 1) (see Table S5 for specific scenario descriptions). The selection of scenario indicators mainly referred to the survey of the Shenzhen Metro Corporation, the Action Plan for Carbon Peaking Before 2030 [45], the 14th Five-Year Plan for Building Energy Efficiency and Green Building Development [46], Shenzhen Comprehensive Transportation “14th Five-Year Plan” [47], etc.

## 3. Results

### 3.1. Energy Consumption Efficiency and Carbon Emission Characteristics of the Metro in the Operational Stage

**Figure 4:**
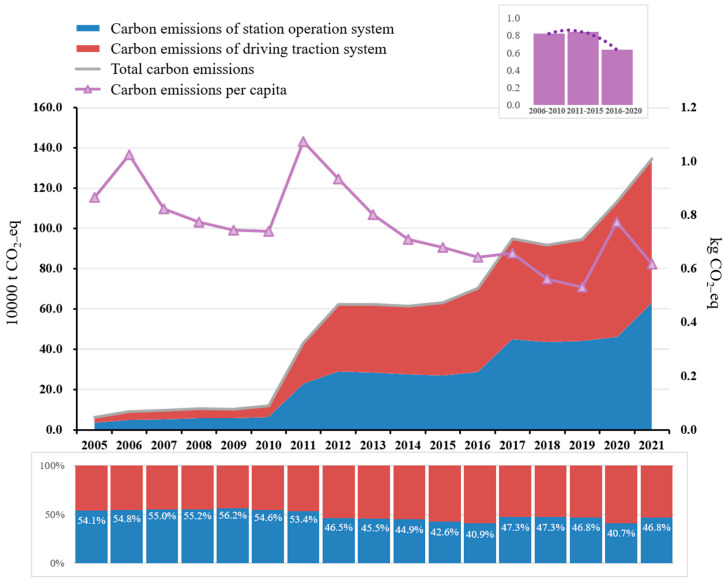
Carbon emissions and proportion of Shenzhen Metro operation from 2005 to 2021.

The carbon emissions of the metro during operation are positively correlated with the intensity of electricity consumption. As shown in Figure 4, from 2005 to 2021, the carbon emissions of the metro operations showed a step-by-step growth, and its comprehensive energy consumption and carbon emissions increased from 60 million kWh (63,000 t CO_2_e) in 2005 to 1.72 billion kWh (1.3 Mt CO_2_e); in other words, carbon emissions increased by a multiple of about 21. The historically accumulated carbon emissions of the Shenzhen Metro are 9.5 Mt CO_2_e. From 2005 to 2021, the carbon emissions of the metro station operation system and the driving traction system accounted for 49% and 51%, respectively. It can be seen from Figure 4 that the carbon emissions of the driving traction system and the station operation system both surged in the same years. For example, the carbon emissions of the driving traction system and the station operation system increased from 54,000 t CO_2_e and 65,000 t CO_2_e in 2010 to 333,000 t CO_2_e and 289,000 t CO_2_e in 2012, respectively. According to the analysis, the carbon emission factor of electricity from 2005 to 2021 showed little change from one year to the next. The reason for the significant increases in carbon emissions during these years is the increases in the operating mileage and number of stations of the Shenzhen Metro, with the opening of new metro lines and extension lines in 2011, 2016, and 2020 (as shown in Figure 1). The energy consumption of a driving traction system increases with the increase in metro operating mileage, and the area devoted to stations increases as more stations are added, so more energy is consumed in maintaining the temperature and humidity of the station space, resulting in a significant increase in the total energy consumption of the metro operation.

**Figure 5:**
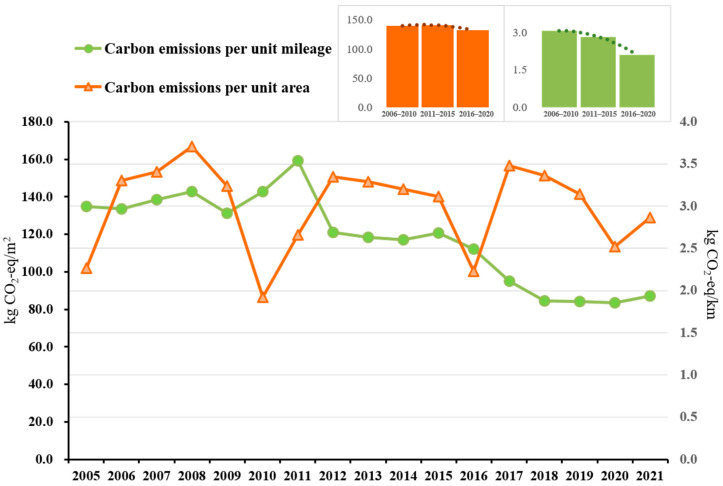
Carbon emissions per unit operating mileage and per unit area of Shenzhen Metro from 2005 to 2021.

The overall trend of carbon emissions per unit operating mileage of the Shenzhen Metro remained relatively stable during 2005–2021 (as shown in Figure 5). The increase in 2011 was due to the opening of new routes. After 2016, due to the impact of energy conservation and emission-reduction policies, the carbon emissions per unit operating mileage of the metro decreased. In order to eliminate the impact of fluctuations in specific years, this study took the five-year emission reduction effect as the benchmark unit and the “13th Five-Year Plan Period” as the reference. The energy consumption and carbon emissions per unit operating mileage of the Shenzhen Metro are 2.5 kWh/km (2.1 kg CO_2_e/km) (see Figure S8 for energy consumption). Compared with the “11th Five-Year Plan Period”, the carbon emissions per unit mileage have been reduced by about 32%. From 2005 to 2021, the carbon emissions per unit area of Shenzhen Metro Station fluctuated greatly (as shown in Figure 5), and there were “troughs” in 2011, 2016, and 2020 due to the large-scale opening of new lines, leading to an increase in the total station construction area. For example, from the end of 2010 to the middle of 2011, the number of metro stations increased from 22 to 46, essentially doubling. In the five-year base period, the carbon emissions per unit area of the station were relatively stable, while energy consumption continued to grow (see Figure S9). The energy consumption and carbon emissions per unit area of Shenzhen Metro stations in the “13th Five-Year Plan Period” were 161.4 kWh/m^2^ (132.5 kg CO_2_e/m^2^), respectively.

In addition, passenger volume has an impact on the energy consumption of metro operations. An increase in passenger volume will induce demand for more metro services, leading to an increase in the number of trains and the frequency of the operation of the station equipment (gates, elevators, etc.). Passenger volume will also affect the passenger-carrying rate of the trains: the traction energy consumption when the train is fully loaded or overloaded is about 5.7% higher than when there are few passengers [48]. This increased usage will also affect the carbon emissions of metro operations. Indeed, the advantages of high capacity and punctuality have resulted in a year-by-year increasing trend in the passenger volume of the metro (as shown in Figure 1); the public transportation share of the Shenzhen Metro line increased from 31% [49] in 2015 to 48.6% [50] in 2020. The COVID-19 pandemic did, of course, affect the volume of metro traffic in 2020; it dropped by about 20% (about 300 million passengers) compared with 2019. Yet, despite this temporary decrease, with the improvement of the Shenzhen Metro line network, its passenger traffic volume has maintained an overall steady growth trend. It can be seen from Figure 4 and Figure S10 that the per capita carbon emissions of the Shenzhen Metro showed a downward trend from 2005 to 2021, and the passenger volume has continued to increase year by year. In response, its energy consumption and carbon emissions decreased from 0.9 kWh per capita (0.8 kg CO_2_e per capita) during the “12th Five-Year Plan Period” to 0.8 kWh per capita (0.6 kg CO_2_e per capita) during the “13th Five-Year Plan Period”. However, these trends were not steady: there were fluctuations in 2011 and 2020 due to the influences of external factors. In 2011, new lines were opened, and extension lines were added, increasing the operating mileage, the number of stations, and passenger volume by 3.4 times, 2.4 times, and 2.8 times, respectively, over those values for 2010. Additionally, electricity consumption grew faster than passenger volume in 2011. In late 2020, although the metro operation mileage reached 411.5 km and the number of stations reached 242, the COVID-19-induced drop in passenger volume caused the per capita energy consumption and the carbon emissions of the Shenzhen Metro operation to increase.

### 3.2. Analysis of the Carbon Emission Reduction Potential of the Metro Operational Stage

**Figure 6:**
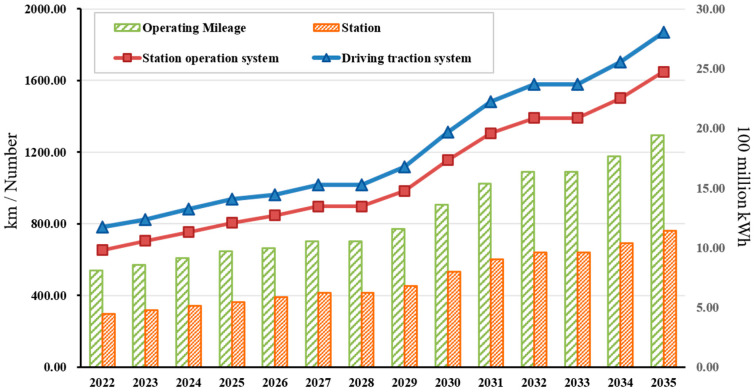
Forecast of Shenzhen Metro operation from 2022 to 2035.

Based on Shenzhen Rail Transit Network Planning (2016–2035), Shenzhen Urban Rail Transit Phase IV Construction Planning, and its adjustment scheme (2017–2022), this research characterized the general situation of Shenzhen Metro planning and construction lines in the short term, medium term, and long term and then forecast the operating mileage and the number and scale of stations in the Shenzhen Metro system over the period of 2022–2035. Then, based on the electricity consumption per unit mileage and per unit station area, the electricity consumption required for metro operation in the future was estimated (as shown in Figure 6). On this basis, we then discussed the effectiveness of energy-saving and emission-reduction measures and analyzed and evaluated the carbon emission reduction potential of these measures.

Shenzhen attaches great importance to exploring the carbon emission reduction potential of the metro system from a technical perspective, such as LED energy-saving lamps, inverter air conditioners, variable-frequency escalators, regenerative braking energy recovery devices, photovoltaic electricity generation systems, etc., which have been gradually promoted and introduced. Among them, the refitting of the public lighting system of the metro stations with LED lighting improved the electricity-saving rate by 58.7%. Furthermore, inverter air conditioning and ventilation-frequency conversion energy-saving technology are used for effective temperature regulation and control, which can achieve “different temperatures for the same vehicle” and promote energy conservation and emission reduction. Moreover, the traction power supply systems of the metro line adopt regenerative braking energy recovery devices, and each line recovers about 4 million kWh annually. The promotion of energy conservation and emission reduction measures has great potential for energy conservation and emission reduction. It is worth noting that Shenzhen is located at 114.05° E and 22.38° N, with an annual average horizontal irradiance of about 1307 kWh/m^2^, which is defined as a Class III solar radiation level, and is rich in solar energy resources (see Table 2). Therefore, the installation of distributed photovoltaic electricity generation systems on the steel structure roofs of the stations and depots is suitable for Shenzen. In this study, roofing monocrystalline silicon solar photovoltaic panels (PV modules) used in Shenzhen Metro Line 6 were used as scenario analysis indicators to predict the carbon emission reduction potential of PV systems. A roof monocrystalline silicon solar photovoltaic electricity generation panel can feed the electrical energy converted from solar energy to the photovoltaic inverter in the photovoltaic equipment room on the platform layer through DC cables and then conduct AC/DC conversion to convert it into 380 V AC, which is then incorporated into the 0.4kV low-voltage switchgear in the substation of the metro station after being connected to the AC grid cabinet. Each station generates about 460,000 kWh of electricity every year. The electrical energy generated by the photovoltaic electricity generation system could supply all low-voltage power loads for metro station lighting, air conditioning, escalators, and other stations. When the electricity generation capacity is insufficient or cannot be generated, it will be supplemented by the metro electricity supply system, which has great potential for energy conservation and carbon reduction.

**Figure 7:**
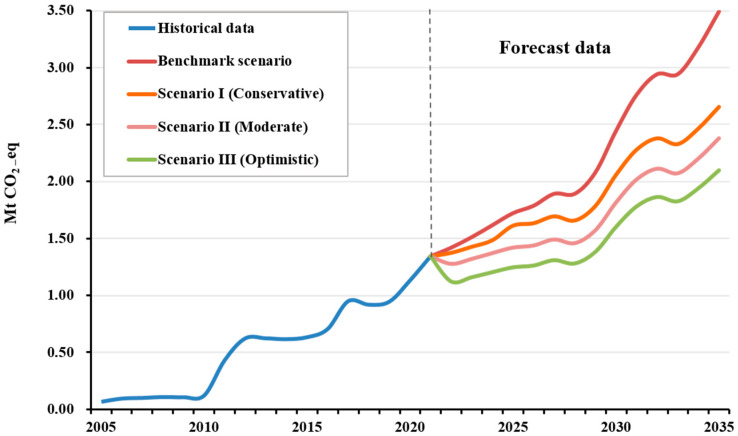
Carbon emission operational-stage prediction values of Shenzhen Metro.

The predicted carbon emissions and emission reduction potential in the operational stage of Shenzhen Metro are shown in Figure 7. The carbon emission trends during metro operation are closely related to the operation mileage and the number of stations. During the entire period of 2005–2021, the overall development of the Shenzhen Metro has maintained an expanding trend, and the operation of the Shenzhen Metro is predicted to continue to grow in the future under all of the four scenarios. It can be seen from Figure 7 that under the benchmark scenario—maintaining the existing energy-saving efforts and the speed of ongoing construction and opening of new metro lines—the carbon emissions during the operational stages of the Shenzhen Metro are expected to increase from 1.34 Mt CO_2_e in 2021 to 3.49 Mt CO_2_e in 2035—an increase of about 2.6 times. In 2025, the carbon emissions of the metro operation under this scenario will be 1.72 Mt CO_2_e.

Compared with the benchmark scenario, the 2025 carbon emissions of Scenario I (Conservative), Scenario II (Moderate), and Scenario III (Optimistic) are estimated to be 1.61, 1.42, and 1.25 Mt CO_2_e, respectively, and the carbon emission reductions are estimated to be 0.11, 0.3, and 0.48 Mt CO_2_e, with decreases of 6%, 21%, and 38%, respectively.

Compared with the benchmark scenario, Scenario I (Conservative) improves the proportion of energy-saving measures and alleviates the growth trend of carbon emissions to a certain extent. In 2035, the carbon emissions of Scenario I (Conservative) are expected to be 2.65 Mt CO_2_e. Compared with the benchmark scenario, the carbon emission reductions are 0.83 Mt CO_2_e, a decrease of about 24%. Scenario II (Moderate) is better than either Scenario I (Conservative) or the benchmark scenario in terms of both energy conservation and emissions reduction; the carbon emissions of Scenario II (Moderate) in 2035 are expected to be 2.38 Mt CO_2_e, about 1.11 Mt CO_2_e lower than in the benchmark scenario, a decrease of about 32%. Scenario III (Optimistic) has the highest proportion of energy-saving measures of all the scenarios; the carbon emissions in 2035 are estimated to be 2.10 Mt CO_2_e, a carbon emission reduction of 1.39 Mt CO_2_e, or 40%, compared to the benchmark scenario value.

In summary, compared to the benchmark scenario, Scenario I (Conservative), Scenario II (Moderate), and Scenario III (Optimistic) can all achieve some degree of carbon emission reduction by implementing energy-saving measures and reasonably optimizing and continuously promoting energy-saving and emission-reduction schemes for the Shenzhen Metro operation, helping to control the growth of carbon emissions.

## 4. Discussion

### 4.1. Comparison of Urban Public Transportation Modes in Shenzhen

In addition to the metro, buses and taxis are the other main types of urban public transportation in China, as reflected in the annual total passenger flow data for various vehicles from 2010 to 2021, which can be found in the monthly statistical report of Shenzhen transportation operation indicators (2010–2021) [42]. At the macro scale, we can compare the carbon emissions generated by the three modes of transportation in the operational process and the accounting methods for bus and taxi carbon emissions, as shown in Equation (2).

(2) $$CE_{PT\left(i\right)}^{t}=N_{i}^{t}\times M_{i}^{t}$$

where t is the type of transportation mode, i.e., bus or taxi; $CE_{PT\left(i\right)}^{t}$ is the carbon emissions of transportation mode t in year i (CO_2_e); $N_{i}^{t}$ is the passenger volume of transportation mode t in year i (100 million passengers); and $M_{i}^{t}$ is the per capita carbon emissions of transportation mode t in year i (kg CO_2_e per capita). Taking the regional characteristics into consideration, this study adopted the local per capita carbon emission index of Shenzhen; that is, the emissions from buses and taxis are 0.54 kg CO_2_e per capita [29] and 1.38 kg CO_2_e per capita [51], respectively.

From the perspective of the carbon emission intensity of the Shenzhen public transportation system, the per capita carbon emissions of the metro and bus networks are 0.6 kg CO_2_e per capita and 0.54 kg CO_2_e per capita, respectively. The per capita carbon emissions of the metro are slightly higher than those of the bus network, and the per capita carbon emissions of taxis are about 2.5 times those of the metro or bus networks; these are similar to the passenger volume and carrying rate of rental cars. According to the existing literature [29,51] and the research results in Section 2.1, the carbon emissions of 1 km of metro operation are 2.1 kg CO_2_e/km (not including the carbon emissions of metro stations), which is much higher than those of the bus network (1.1 kg CO_2_e/km) or taxis (0.3 kg CO_2_e/km), because the metro operating environment, carrying space, and other factors require high electricity consumption per km of metro operation, resulting in increased carbon emissions.

**Figure 8:**
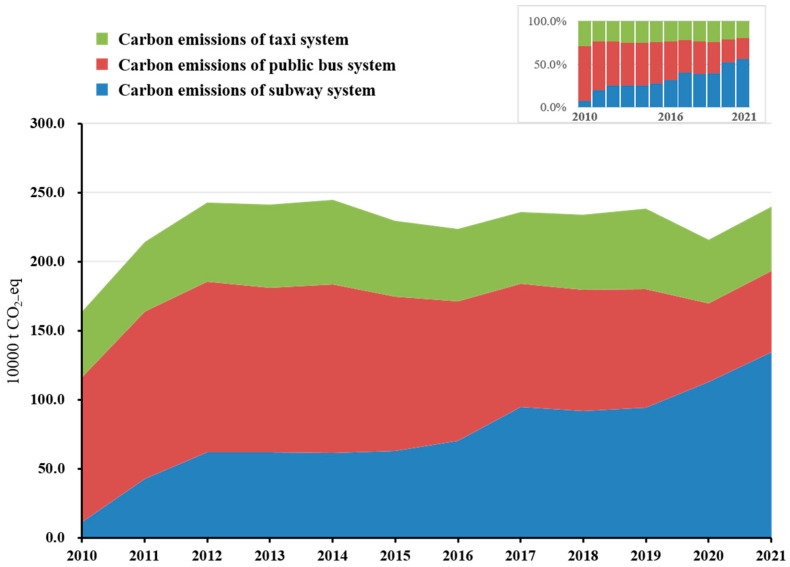
Carbon emissions of Shenzhen public transportation system from 2010 to 2021.

The carbon emissions from the three main types of urban public transportation from 2010 to 2021 are shown in Figure 8. It can be seen that the overall trend of carbon emissions from Shenzhen’s public transportation system is relatively stable. From 2011 to 2021, the total carbon emissions of Shenzhen’s public transportation remained above 2 Mt and reached a peak of 2.5 Mt in 2014, with cumulative carbon emissions of 27.2 Mt CO_2_e. In general, the carbon emissions of bus transportation have been declining year by year over the past decade for two reasons: Shenzhen Metro line’s increased share of passenger volume (Shenzhen Metro, 2021) has come at the expense of a drop in bus passenger volume and hence in the decreased operation of buses; and the bus system has been investing in new, more efficient, and greener types of energy [29]. The overall carbon emissions of taxi transportation have been relatively stable; the fluctuation of carbon emissions has remained within 10%. In addition, the proportion of carbon emissions contributed by the metro, of the emissions from all types of public transportation, increased from 7.2% in 2010 to 56.1% in 2021, with an average annual growth rate of 4.4%, while the emissions from buses decreased from 63.9% in 2010 to 24.6% in 2021 and those of taxis varied between 19.3% and 28.9%. Before 2016, bus transportation was the main source of carbon emissions from the urban public transportation system, but after 2016, the passenger travel structure changed, and the metro began to dominate. At the same time, the COVID-19 pandemic impacted bus and taxi transportation as well as metro operation, reducing passenger volume.

The metro, new green-energy buses, and taxis are generally considered to be green transportation modes because they generate no direct emissions during their operational stage. However, according to the above calculation results, the operation of the urban public transportation system, especially the metro, will consume a great deal of electricity, and thus the metro has gradually become the main contributor of emissions from urban public transportation. Therefore, considering the upstream production stage of electricity, the metro, green-energy buses, and taxis are not necessarily absolutely green transportation modes because the electricity used in Shenzhen does not come entirely from clean energy [52]. The environmental impacts caused by the large consumption of electricity will, therefore, also increase. To realize a true green and low-carbon transformation and the development of urban public transportation in Shenzhen, it is necessary to further improve the energy utilization efficiency and the proportion of clean energy and strengthen the implementation of energy-saving technologies.

### 4.2. Comparison of Metro Operational Stage in Different Cities

**Figure 9:**
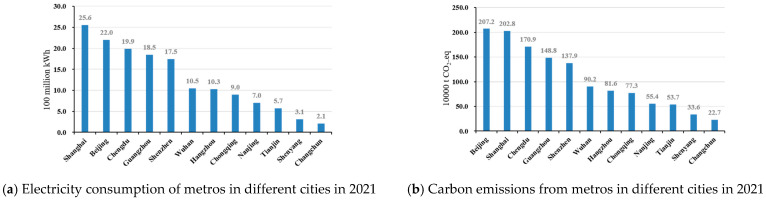

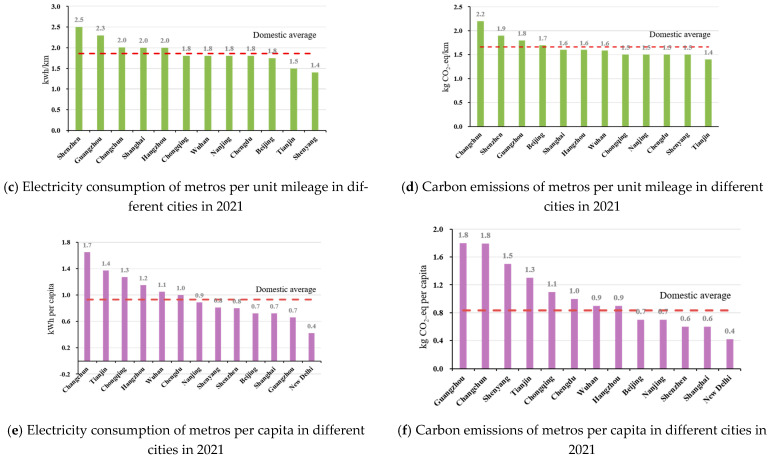
Energy consumption and carbon emission intensity of metro operation in different cities in 2021.

Metros are an important part of an urban public transportation system and one of the main sources of carbon emissions (for example, the total carbon emissions from metro operations in Shenzhen accounted for 56.1% in 2021). In this study, the results of energy consumption and carbon emission intensity during metro operations in Shenzhen in 2021 were compared with those from some domestic cities (as shown in Figure 9). A total of 12 domestic cities were selected for comparison; these included four first-tier cities (Shanghai, Beijing, Guangzhou, and Shenzhen) and urban areas in East China, South China, North China, Central China, Northwest China, and Northeast China. See Table 3 for the metro information of the selected cities.

Overall, in 2021, the Chinese national metro operation consumed 21.3 billion kWh of electricity, and the total carbon emissions were about 19.1 Mt CO_2_e. As can be seen from Figure 9a,b, Beijing, Shanghai, and Guangzhou were the top three cities for China’s energy consumption and carbon emissions, accounting for more than 30% of the country’s totals. Although the power consumption in Shanghai was higher than that in Beijing, the carbon emissions in different regions were reversed due to the differences in the power carbon emission factors. As for energy consumption and carbon emission intensity of metro operation, this study compared these figures per unit operating mileage with per capita energy consumption and carbon emission indicators. As shown in Figure 9c,d, in 2021, the national average electricity consumption per unit operating mileage was 1.9 kWh/km, and a total of five of the cities studied had values higher than the national average. Among these, Shenzhen and Guangzhou were the largest, with 2.5 kWh/km and 2.3 kWh/km, respectively, and Changchun was the third. As for the carbon emissions per unit mileage, only three cities exceeded the national average value of 1.7 kg CO_2_e/km, with Shenzhen ranking second after Changchun. The energy consumption and carbon emissions per unit operating mileage are directly related to the traction electricity consumption and the operating mileage of the trains. The geographical layout of the metro lines, the vehicle weight, the use of energy-saving technology and other factors all affect the energy consumption and carbon emissions of a train. The passenger volume also affects the number of metro trains, as well as the operating mileage. For per capita energy consumption and carbon emissions, as shown in Figure 9e,f, the per capita electricity consumption of the Shenzhen Metro was lower than the national average of 0.9 kWh per capita; Changchun, Tianjin, and Chongqing occupied the top three in China. Comparing the per capita carbon emissions, Shenzhen’s emissions in 2021 were 0.6 kg CO_2_e/km, slightly lower than the national average (0.8 kg CO_2_e/km). The per capita energy consumption and carbon emissions are directly related to the total electricity consumption and passenger volume of a metro. The size of the metro system, in turn, affects the total energy consumption, and the passenger volume is also affected by the geographical layout of the lines.

From the comprehensive data shown in Figure 9, looking at all the 12 cities studied, it can be seen that Shenzhen Metro’s operation still has significant room for improvement in terms of carbon emission and energy consumption reduction. By contrast, though, Nanjing is performing better in both aspects. Shenzhen Metro’s per unit operating mileage energy consumption and carbon emissions, and per capita energy consumption and carbon emissions all show downward trends (see Figure 3 and Figure 4 for details), indicating that the metro will play a vital role in reducing the carbon emissions from urban public transportation systems in the future. Therefore, the Shenzhen Metro can make a major contribution to reducing carbon emissions by investing in energy-saving and emission-reduction technology, while the electrical system can aid this effort by increasing its proportion of clean new energy (such as wind power, photovoltaic, geothermal energy, etc.).

### 4.3. Suggestions

With the development of the Guangdong–Hong Kong–Macao Greater Bay Area, the metro, as an important tool for solving urban traffic congestion, will continue to increase its number of stations, vehicle and passenger volumes, and operating mileage [54]. The carbon emissions behind the energy consumption of metro operations will also increase year by year. In this regard, formulating and implementing relevant policies and standards and actively taking energy-saving and emission-reduction measures will help to slow the growth of the carbon emissions of the Shenzhen Metro and promote the low-carbon transformation and development of urban public transportation in Shenzhen. Based on the findings of this study, the following reasonable recommendations are made.

(1) Formulate and implement relevant energy conservation and emission-reduction policies and standards in the field of urban public transportation. In terms of energy conservation and emission reduction, Shenzhen has made great efforts to build green-energy infrastructure and promote energy conservation, such as the Shenzhen Green Building Promotion Measures and the Shenzhen Special Economic Zone Green Building Regulations. However, there are no relevant policies or measures for energy conservation and emission reduction in the transportation field. Construction and transportation have always been high resource- and energy-consuming industries, and the environmental burden they bring to society cannot be ignored. Therefore, the introduction of energy-saving and emission-reduction policies and standards is necessary, and they are guaranteed to promote the low-carbon transformation of urban public transportation systems.
(2) Reasonably plan the layouts of metro lines and improve the efficiency of metro transportation. Compared with foreign cities such as London and New York, metro construction in China started late, beginning in Shenzhen, for example, in the late 1990s. The study results show that the per capita carbon emissions of the Shenzhen Metro (0.6 kg CO_2_e) are similar to those of buses (0.54 kg CO_2_e) and are much lower than those of taxis (1.38 kg CO_2_e). Furthermore, the per capita carbon emissions curve of the metro shows a downward trend, indicating that the metro will play a vital role in reducing carbon emissions from urban public transportation in the future. In addition, at present, the full load rate of the Shenzhen Metro is relatively low. If the metro line layout can be well aligned with people’s travel needs, the metro’s share of passenger traffic can be further improved. Taking Tokyo, Japan, as an example, the construction of a compact metro is conducive to the sustainable development of a city [55]. Therefore, the Shenzhen Metro can learn from the mature experience of Tokyo and other cities, building metro systems to cover several different service levels; taking into consideration the area to be served, the travel demand characteristics, and travel time; improving the interconnection of metro lines at different service levels; and promoting the formation of an integrated metro network.
(3) Optimize the energy structure, improve the efficiency of thermal power generation and clean transformation, increase the proportion of new clean energy, and realize the zero-carbon transformation of energy. Although the electricity consumed by metro operations does not directly produce greenhouse gases, metro energy utilization can be improved, and the upstream production of this electricity also needs to be considered. Optimizing the energy structure can reduce emissions by up to 32% per year, with a reduction of about 1.35 Mt CO_2_e (Scenario III in 2035). Clean energy generation plays an important role in the low-carbon transformation of transportation. In particular, photovoltaic systems can be widely used on the roofs of stations and depots to share energy consumption for the operation of station lighting systems. The photovoltaic electricity generation system can generate up to 47.106 million kWh of electricity per year, and the emission reduction is about 289,000 tCO_2_e (Scenario III in 2035). In the future, more clean energy technologies should be explored, especially photovoltaic electricity generation systems (such as perovskite photovoltaic (PV) cells) [56,57], to introduce greater carbon emission reduction benefits to the subway system.
(4) Promote energy-saving and emission-reduction technologies and schemes. The result shows that the energy-saving equipment used in Shenzhen’s Metro (frequency-conversion air conditioners, LED energy-saving lamps, frequency-conversion escalators, etc.), as well as photovoltaic electricity generation technology and regenerative braking energy recovery technology (realized for the first time in Shenzhen on the metro line), can achieve a significant carbon emission reduction effect. The research results show that photovoltaic electricity generation technology can generate 50 million kWh per year, regenerative braking energy recovery technology can recover 190 million kWh per year, and energy-saving lamps, frequency-conversion air conditioners and frequency-conversion escalators can save 1.18 billion kWh per year (Scenario III in 2035). However, since energy-saving and emission-reduction technology have not yet been fully implemented in the Shenzhen Metro, the use of emission-reduction technology should be further expanded in the future.

### 4.4. Constraints and Limitations

The methods proposed in this study and the quality of the inventory data used have some limitations. First, the current analysis is limited to energy consumption and carbon emissions and lacks consideration of the end-point environmental impacts. In future research, the terminal environmental impacts under regional characteristics should be discussed in order to fully understand the environmental characteristics of the metro. Secondly, for the statistical data on the energy consumption of the metro system, the influence of uncertain factors has not been considered, and more accurate base statistics should be obtained. Then, the carbon emission factor of electricity generation should be selected to be as close as possible to the China Southern Power Grid in Shenzhen. Finally, the boundary of the research system of this study was relatively narrow, considering only the operational stage of the metro, and the calculation method only considered the per capita carbon emissions due to data unavailability. The design and construction stages should be taken into account in future studies.

## 5. Conclusions

With the rapid expansion of the transportation industry and the growth of urban populations, the low-carbon development of urban public transport systems faces major challenges. Metro operation not only consumes a lot of energy but also intensifies potential environmental risks. Based on the LCA method, this study constructed a carbon emission calculation method for the operation phase of the metro system, comprehensively evaluated the carbon emission level and the effectiveness of energy conservation and emission reduction during the operational phase of the Shenzhen Metro from two aspects, station lighting systems and the driving traction, and explored its characteristics from three aspects: station area, traffic traction, per capita energy consumption, and carbon emission intensity. At the same time, other public transportation modes and the metro operation of typical domestic cities were compared and analyzed so as to provide support for green, low-carbon transformation in the transport field and the formulation of energy-saving management policies for the metro system. The main contributions of this study were: (1) the results of the life-cycle analysis show that the total carbon emissions of Shenzhen Metro operation maintained continuous growth from 2005 to 2021, from 63,000 t CO_2_e in 2005 to 1.3 Mt CO_2_e in 2021, and the historically accumulated carbon emissions were 9.5 Mt CO_2_e. (2) The overall energy consumption and carbon emission intensity of the Shenzhen Metro both show a downward trend (except for the energy consumption per unit station area), but it has achieved no advantage compared to the national average levels, and there is still room for improvement in both emission and consumption reduction. (3) The Shenzhen Metro has gradually become the main contributor to carbon emissions in the urban public transportation system. In 2021, the carbon emissions of Shenzhen Metro operation accounted for about 56.1% of the total emissions from the urban public transportation system. (4) An advanced low-carbon model (from conservative to optimistic scenarios) can significantly alleviate the rapid growth of carbon emissions of Shenzhen Metro. The maximum carbon emission reduction rate could reach 40% per year (2035), and the cumulative carbon savings from 2022 to 2035 could reach 0.1 Mt CO_2_e. Therefore, in order to realize a green and low-carbon transformation in the field of transportation, large cities should optimize their energy structure as soon as possible, increase the proportion of clean energy generation, and realize a zero-carbon transformation of energy use, while also further promoting energy-saving and emission-reduction technologies and schemes, especially in their metro systems. In addition, megacities should prioritize the development of metros and plan the layout of metro lines to maximize their use and their transportation efficiency. The methods and results of this study can provide a reference for the development of low-carbon transportation systems in other megacities in China.

## Figures and Tables

**Table 1:** Scenario analysis index setting of Shenzhen Metro operational stage.

Indicator	Benchmark Scenario		Scenario I (Conservative)		Scenario II (Moderate)		Scenario III (Optimistic)	
	2025	2035	2025	2035	2025	2035	2025	2035
Photovoltaic electricity generation	28%	28%	39%	60%	48%	80%	54%	90%
Regenerative braking energy recovery	3%	3%	12%	30%	21%	50%	34%	80%
LED energy-saving lamps	89%	89%	97%	100%	100%	100%	100%	100%
Inverter air conditioners	65%	65%	82%	100%	94%	100%	100%	100%
Variable-frequency escalators	90%	90%	94%	100%	100%	100%	100%	100%
Electricity carbon emission factor (kg CO_2_e/kWh)	0.799	0.799	0.784	0.664	0.713	0.604	0.641	0.543

Notes: The carbon emission factor forecast for electricity is shown in Tables S1 and S4–S6.

**Table 2:** Energy level division table of solar energy resources.

Level	Resource Code	Annual Total Radiation (MJ/m^2^)	Annual Total Radiation (kWh/m^2^)	Average Daily Radiation (kWh/m^2^)
Zone of maximum abundance	I	≥6300	≥1750	≥4.8
Zone of abundance	II	5040~6300	1400~1750	3.98~4.8
More abundant zone	III	3780~5040	1050~1400	2.9~3.93
General	IV	<3780	<1050	<2.9

**Table 3:** Data for comparison of energy consumption and carbon emissions of metros in 13 cities in 2021.

City	Beijing	Shanghai	Guangzhou	Shenzhen	Chengdu	Wuhan	Chongqing	Nanjing	Hangzhou	Tianjin	Changchun	Shenyang
Lines	23	18	13	12	12	11	7	5	9	7	2	4
Mileage (km)	709.9	795.4	505.7	420.6	518.5	435.3	271	182.2	342	211.8	43	114.1
Stations	390	486	296	247	327	275	208	200	185	179	126	161
Passenger volume (100 million passengers)	30.4	35.6	21.8	21.8	12.1	10.0	7.1	7.9	8.9	4.1	1.3	3.8

Data source: Statistics and Analysis Report of Urban Rail Transit in 2021 [53].

## Data Availability

We have participated sufficiently in work to take public responsibility for the appropriateness of the collection, analysis, and interpretation of the data.

## Supplementary Materials

The following supporting information can be downloaded at: https://www.mdpi.com/article/10.3390/ijerph20010206/s1. References [58,59,60,61,62,63,64] are cited in the Supplementary Materials.
