# Supplementary material for: Uncovering the Carbon Emission Intensity and Reduction Potentials of the Metro Operation Phase: A Case Study in Shenzhen Megacity
Source: Int J Environ Res Public Health. 2022 Dec 23;20(1):206. doi: 10.3390/ijerph20010206 (PMC9819634; doi:10.3390/ijerph20010206)
Supplement: Supplementary file 1 [file ijerph-20-00206-s001.zip › ijerph-2069938-supplementary.pdf]

Supporting information for:

Uncovering the Carbon Emission Intensity and Reduction Potentials of the Metro Operation Phase: A Case Study in Shenzhen Megacity

Kunyang Chen [1,2], Guobin Zhang [3,*], Huanyu Wu [1,2], Ruichang Mao [4] and Xiangsheng Chen [1,2]

[1] Key Laboratory for Resilient Infrastructures of Coastal Cities, Ministry of Education, Underground Polis Academy, College of Civil & Transportation Engineering, Shenzhen University, Shenzhen 518061, China
[2] Shenzhen Key Laboratory of Green, Efficient and Intelligent Construction of Underground Metro Station, Shenzhen University, Shenzhen 518060, China
[3] State Environmental Protection Key Laboratory of Mineral Metallurgical Resources Utilization and Pollution Control, Wuhan University of Science and Technology, Wuhan 43008, China
[4] School of Civil Engineering, Tsinghua University, Beijing 100084, China
* Correspondence: mecgbzhang@126.com

Outline of supporting information

1. Summary of the calculation, formulas and data sources

2. Method, data inventory and calculation

Table S1 Data sources, calculation and formulas.

**Important parameters**		**Formula**	**Unit**	**Data Sources**	**Explanations**
1. Annual energy consumption of subway in operation stage	1.1 Station operation system	$U_S = \sum_{i=1}^{n} U_{S,L}$;	*kwh*	2005-2021:<br>-Annual subway line length, operating mileage, stations and station area: Official website of Shenzhen Metro (2021) (from 2007-2020) and China Association of Metros, see Fig. 1, S2 and S3.<br>-Annual electricity consumption of station power and lighting: Subway operation electricity statistics account from Shenzhen Metro company (Lines 1,2,3,4,5,6,7,8,9,10,11) (from 2005-2020), see Fig. S1.<br>- Annual electricity consumption of driving traction: Shenzhen subway operation energy consumption statistics monthly report from Shenzhen Metro (Lines 1,2,3,4,5,6,7,8,9,10,11) (from 2005-2020) , see Fig. S1.<br>2022-2035:<br>- Projected data on annual subway Line length, opening date, stations: Official website of Shenzhen Metro (2021); Transport Commission of Shenzhen Municipality [58]; Shenzhen Urban Rail Transit Construction Planning (2017-2022) [59]; Shenzhen rail transit network planning (2016-2035) [9], see Fig. S5.<br>- Annual electricity consumption of station power and lighting and driving traction: Our calculation based on the historical data and planning. See Fig. S6.	U is the energy consumption (electricity);<br>S is the station power and lighting;<br>D is the driving traction;<br>The year of n;<br>L is the line of subway
	1.2 Energy consumption of driving traction	$U_D = \sum_{i=1}^{n} U_{D,L}$;			
2. Annual passenger volume	2.1 Annual passenger volume of subway		*100 million passenger*	- Annual passenger volume of subway transport: Shenzhen Metro Group Co., Ltd. Annual Report (2007-2009) of Shenzhen Metro (2021); The transport production statistics monthly report (2010-	See Table S2

	transport			2021) in Transport Commission of Shenzhen Municipality [42].	
	3.2 Annual passenger volume of bus transport		*100 million passenger*	- Annual passenger volume of bus transport: The transport production statistics monthly report (2010-2021) in Transport Commission of Shenzhen Municipality [42].	
	3.3 Annual passenger volume of taxi transport		*100 million passenger*	- Annual passenger volume of taxi transport: The transport production statistics monthly report (2010-2021) in Transport Commission of Shenzhen Municipality [42].	
3. Carbon emission impacts	3.1 Annual carbon emission factors of electricity		*kg CO_2e /kWh*	2005-2021:<br>-Annual carbon emission factors of electricity: National Development and Reform Commission. China regional power grid baseline emission factor (2006-2019) https://www.mee.gov.cn/ *(In Chinese)*<br>2022-2035:<br>- Projected data on annual carbon emission factors of electricity: Our own calculation, based on reported carbon emission estimates for the energy mix (e.g. Thermal power, Hydro, Nuclear power, Wind-Onshore, Solar, Gas), also refer to Zhou et.al (2012) , uncertainty is considered.	See Table S3; the projected data on annual carbon emission factors of electricity see Table S4.1, Table S4.2, Table S4.3

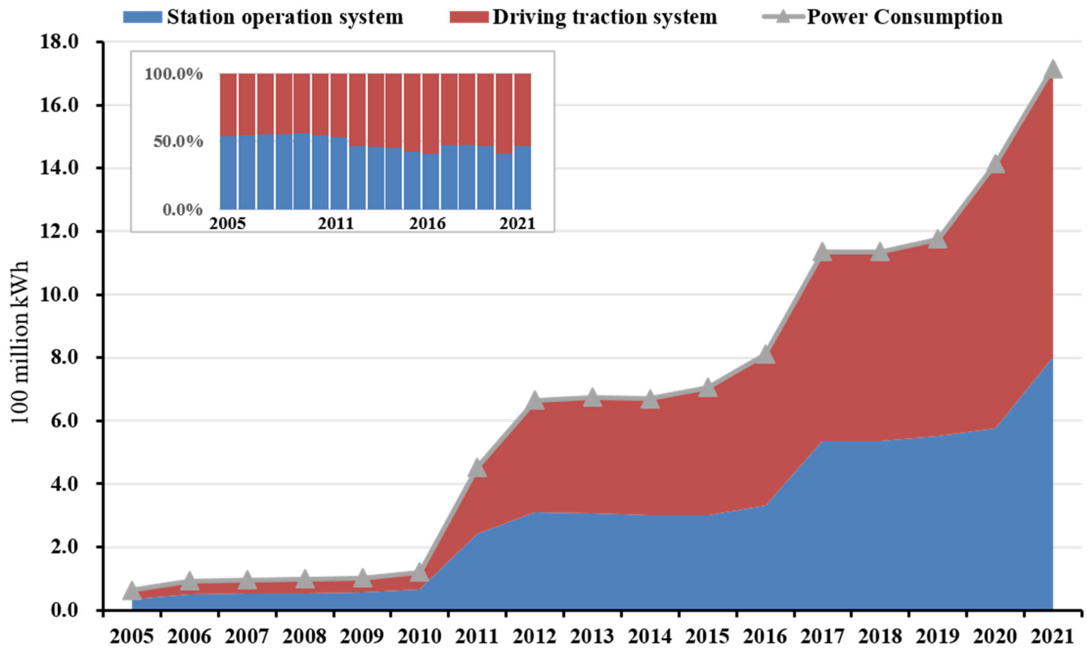

Figure S1 Operational Energy Consumption of Shenzhen Metro from 2005 to 2021.

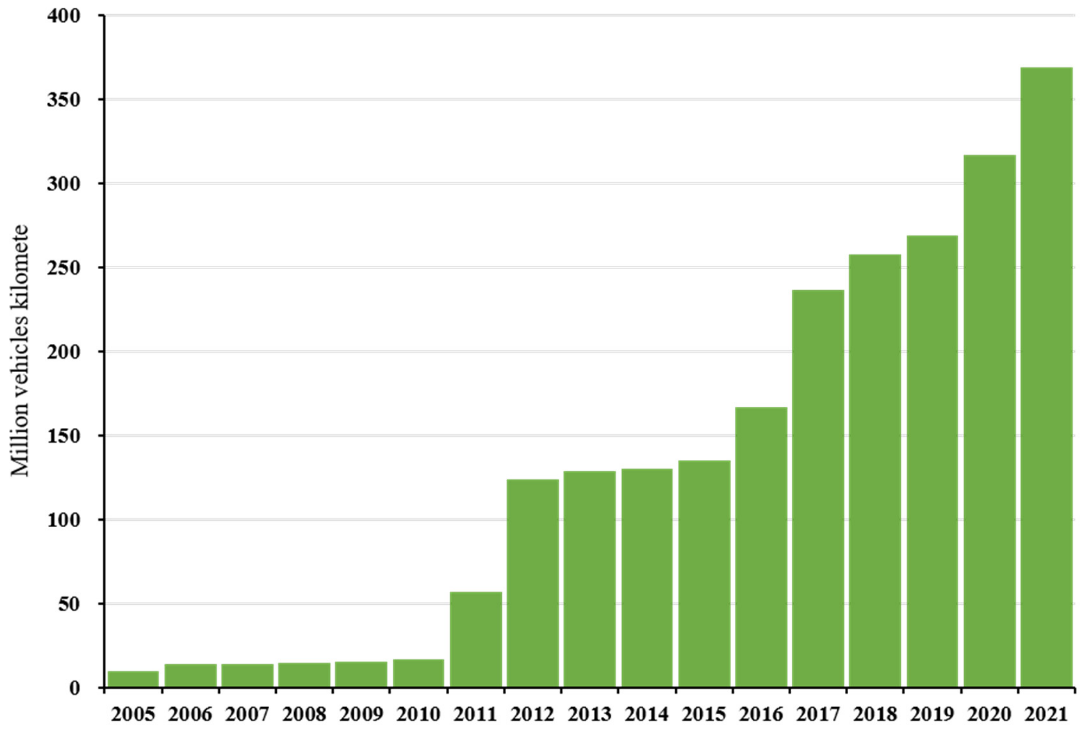

Figure S2 The operating mileage of Shenzhen Metro from 2005 to 2021.

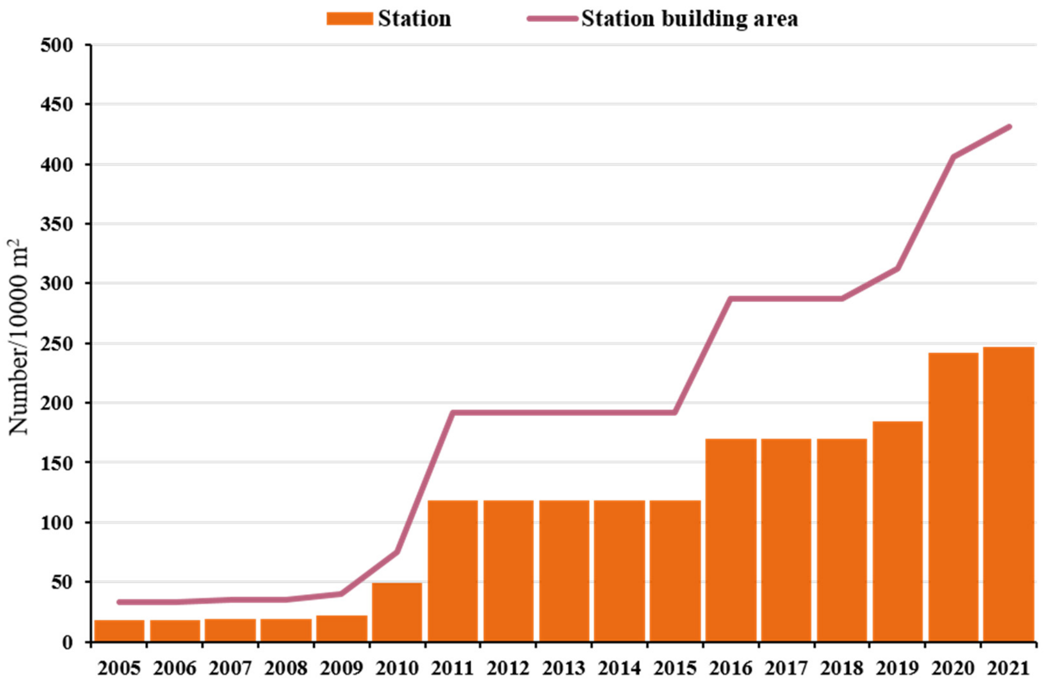

Figure S3 The number and scale of Shenzhen Metro stations from 2005 to 2021.

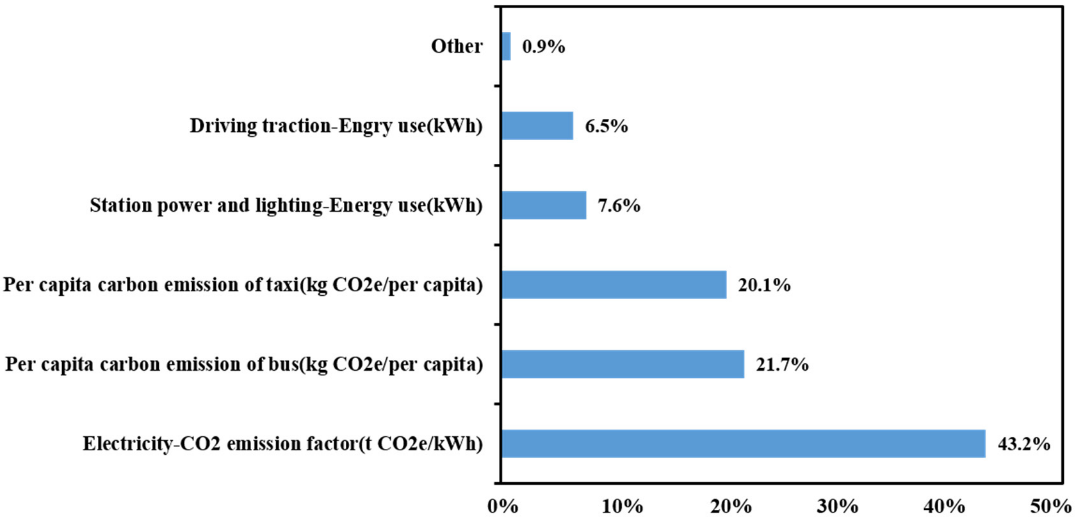

Figure S4. Sensitivity analysis

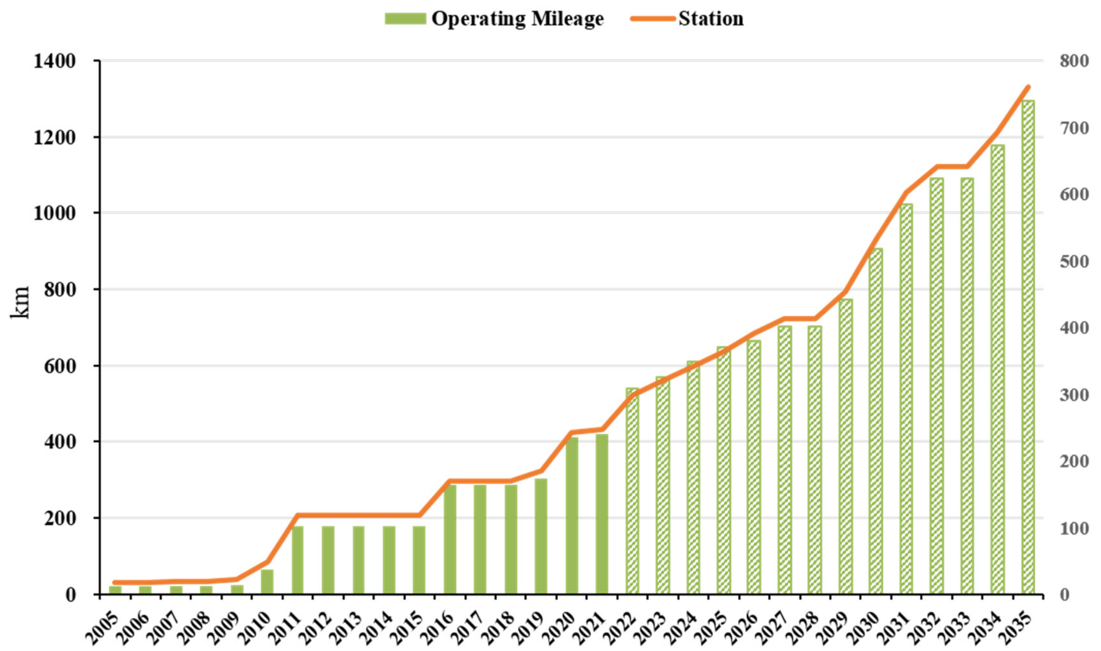

Data sources: Shenzhen Transportation Bureau [42]); Shenzhen Rail Transit Planning (2016-2035); Shenzhen Urban Rail Transit Construction Planning (2017-2022); official website of Shenzhen Metro (2021).

Figure S5 The length and station of Shenzhen Metro in 2022-2035.

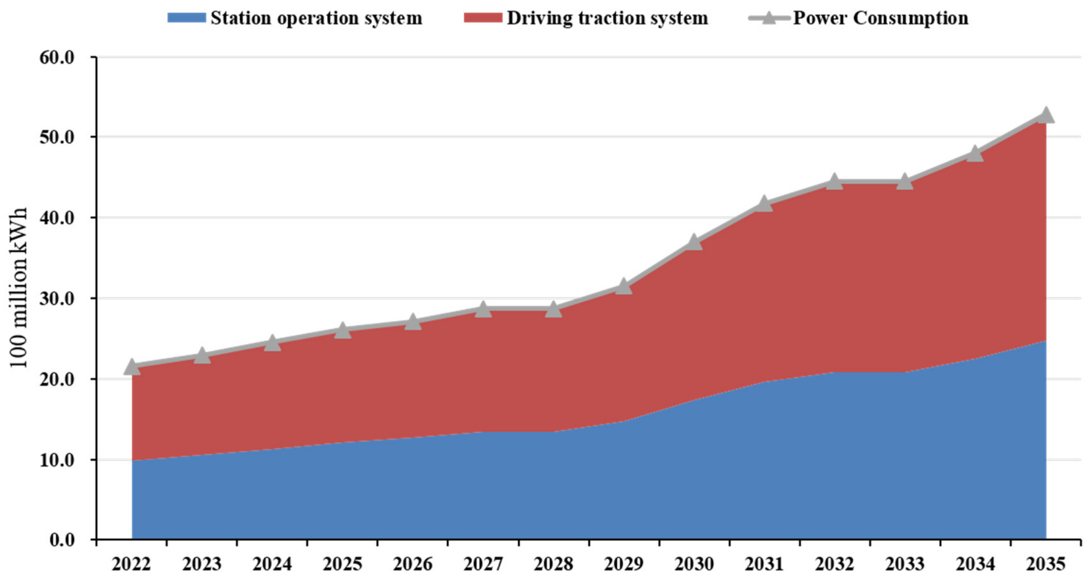

Figure S6 Operational Energy Consumption of Shenzhen Metro from 2022 to 2035.

Table S2 Annual passenger volume of subway, bus and taxi in Shenzhen.

Year	Subway	Bus	Taxi
2010	1.63	19.42	3.38
2011	4.60	22.37	3.63
2012	7.81	22.83	4.08
2013	9.16	22.02	4.32
2014	10.37	22.57	4.38
2015	11.22	20.69	3.91
2016	12.97	18.68	3.74
2017	16.54	16.54	3.71
2018	18.77	16.29	3.88
2019	20.21	15.90	4.13
2020	16.23	10.54	3.27
2021	21.79	10.90	3.31

Table S3 Carbon emission factors of electricity.

Year	Emissions factors	Unit	Geography	Data source [41]
2005	1.005	Kg CO_2e/kWh	China South Power grid	China regional power grid baseline emission factor (2006-2019); 2020-2021 from linear forecast, see Fig. S7
2006	0.985			
2007	1.012			
2008	1.061			
2009	0.999			
2010	0.976			
2011	0.949			
2012	0.934			
2013	0.922			
2014	0.918			
2015	0.896			
2016	0.868			
2017	0.837			
2018	0.809			
2019	0.804			
2020	0.799			
2021	0.784			

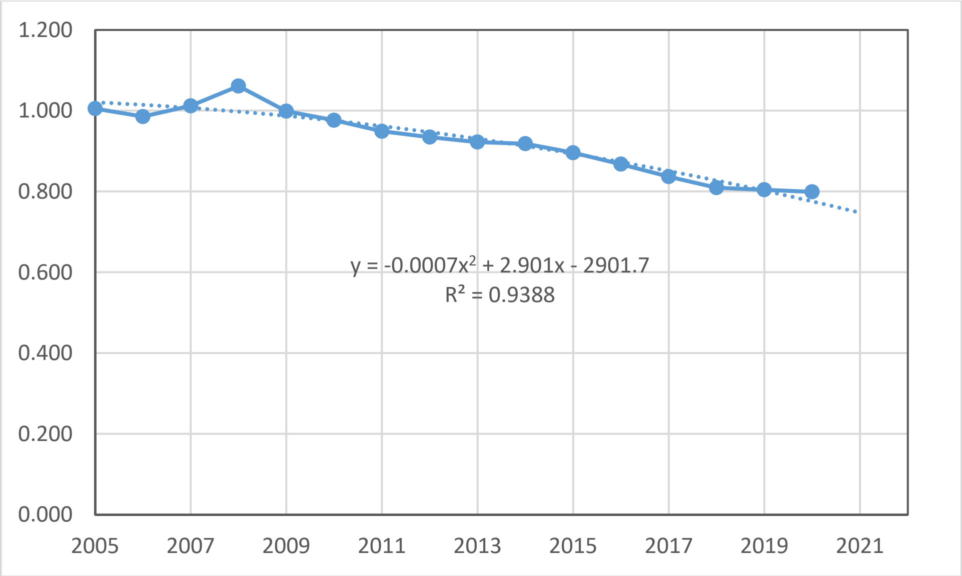

Data sources: China regional power grid baseline emission factor (2006-2019).

Figure S7 Carbon emission factor from 2019 to 2020.

Table S4.1 Reported carbon emission estimates for electricity.

Energy technology	Life cycle CE estimates ($kgCO_2$ e/kWh)	geography	Data source
Thermal power	1.101	China	CLCD
Hydro	0.011	South China	CLCD
Nuclear power	0.012	Global	IPCC 2014
Wind-Onshore	0.009	China	Literature
Solar	0.011	China	Literature
Gas	0.490	Global	IPCC 2014

Data sources: The data of Wind-Onshore are retrieved from [60]; similarly, the data of Solar are drawn from [61].

Table S4.2 Power plant structure.

Year	Thermal power	Hydro	Nuclear power	Wind	Solar	Gas
2014	74.9%	14.4%	7.9%	2.2%	0.6%	0
2020	54.4%	5.6%	12.3%	6.1%	4.6%	16.9%
2030	42.5%	18.8%	5.6%	18.8%	14.6%	0

Data sources: The power plant structure in 2014 is based on the Guangdong Province power plant installed capacity (million kwh) [62]; In 2020, the Power plant structure is estimated by "13th Five - Year Plan for Energy Conservation and Emission Reduction in Guangdong Province"; In 2030, the energy mix is estimated by "China Energy Outlook 2030" report (2016)[63].

Table S4.3 Projected carbon emission factors of electricity in four scenarios.

Year	Unit	B Scenario	S I (Conservative)	S II (Moderate)	S III (Optimistic)
2022	kg CO_2e/kwh	0.784	0.781	0.763	0.687
2023		0.784	0.783	0.746	0.671
2024		0.784	0.765	0.729	0.656
2025		0.784	0.784	0.713	0.641
2026		0.784	0.771	0.701	0.631
2027		0.784	0.758	0.689	0.620
2028		0.784	0.746	0.678	0.610
2029		0.784	0.733	0.667	0.600
2030		0.784	0.721	0.656	0.590
2031		0.784	0.710	0.645	0.581
2032		0.784	0.698	0.634	0.571
2033		0.784	0.686	0.624	0.562
2034		0.784	0.675	0.614	0.552
2035		0.784	0.664	0.604	0.543

Note: Based on the data in Table S4.1, Table S4.2 and Zhou(2012)[64], we can get the decrease rate of carbon emission factors of electricity is 1.5% and 1.2% in 2010-2020 ,2020-2030, respectively. The data of S II is based on the decrease rate of carbon emission factors of electricity above, the data for previous years from China regional power grid baseline emission factor. The data in S I and S III is take as the increase of 10%, -10% on the basis of S II. The data of Business as usual scenario is the same as that of 2021.

Table S5 Description of the four scenarios.

Scenario	Description
Benchmark scenario	The benchmark scenario is a scenario in which the current development model is maintained, without considering the impact of more policy planning and without taking any energy-saving measures other than the status quo. It can provide a relatively intuitive comparison with other scenarios. Under the assumption of the benchmark scenario, the use of various energy-saving measures will remain the same as in 2021. Although inverter air conditioners and energy-saving lamps have been vigorously promoted, and can achieve the development goals of saving energy and reducing emissions during the operation of new subway lines, a certain energy-saving transformation process is still required to cover the entire railway network.
Scenario I (Conservative)	Scenario I (Conservative) is based on the benchmark scenario, which increases the proportion of energy-saving indicators set to different degrees, optimizes the energy structure, increases the proportion of clean new energy, and reduces the carbon emission factor in the upstream production stage of electricity. Compared with other indicators, the penetration rate of photovoltaic power generation and regenerative braking energy recovery is low. Shenzhen Metro Line 6 opened in 2020 is the first large-scale application of distributed photovoltaic power generation in China, and it is also the first subway line with regenerative braking energy recovery in Shenzhen.
Scenario II (Moderate)	In scenario II (Moderate), the implementation of energy-saving and emission reduction policies and standards will be further strengthened. By 2035, the proportion of subway frequency conversion air conditioner, LED energy-saving lamp and frequency conversion escalator will reach 100%, and the application proportion of photovoltaic power generation and regenerative braking energy recovery technology will be increased to 80% and 50% respectively. With the increase of the proportion of clean new energy, as the main energy for subway operation, the carbon emission factor of electric power is reduced to 0.604 kg CO_2e / kWh. Compared with scenario I (conservative), the low-carbon development of subway operation process has been further strengthened.
Scenario III (Optimistic)	Scenario III (Optimistic) is a scenario in which the government and enterprises actively promote energy-saving planning, and the subway energy-saving measures and energy-saving transformation are comprehensively and effectively

implemented. It is the most ideal scenario in all scenarios. Under this scenario, 100% of the subway variable-frequency air conditioner, LED energy-saving lamp and variable-frequency escalator will be applied in 2025. Considering the late start of the application of photovoltaic power generation and regenerative braking energy recovery technology, the recycling of photovoltaic power generation and regenerative braking energy will reach 90% and 80% in 2035, and the electric carbon emission factor will be reduced to 0.543 kg CO_2e / kWh. The low-carbon and green development of subway operation system presents an ideal state.

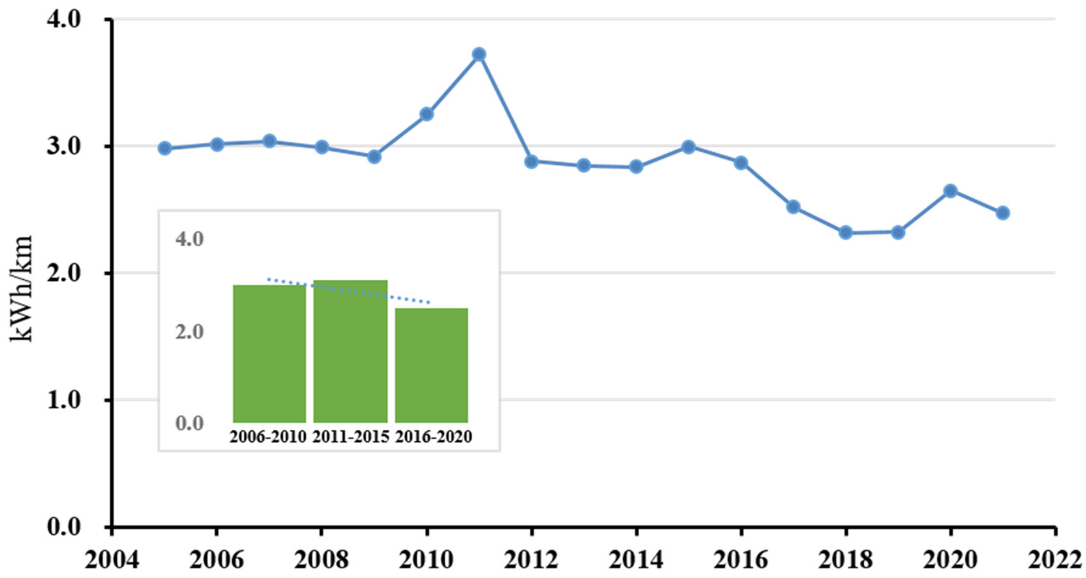

Figure S8 Energy consumption per unit mileage of Shenzhen Metro from 2005 to 2021.

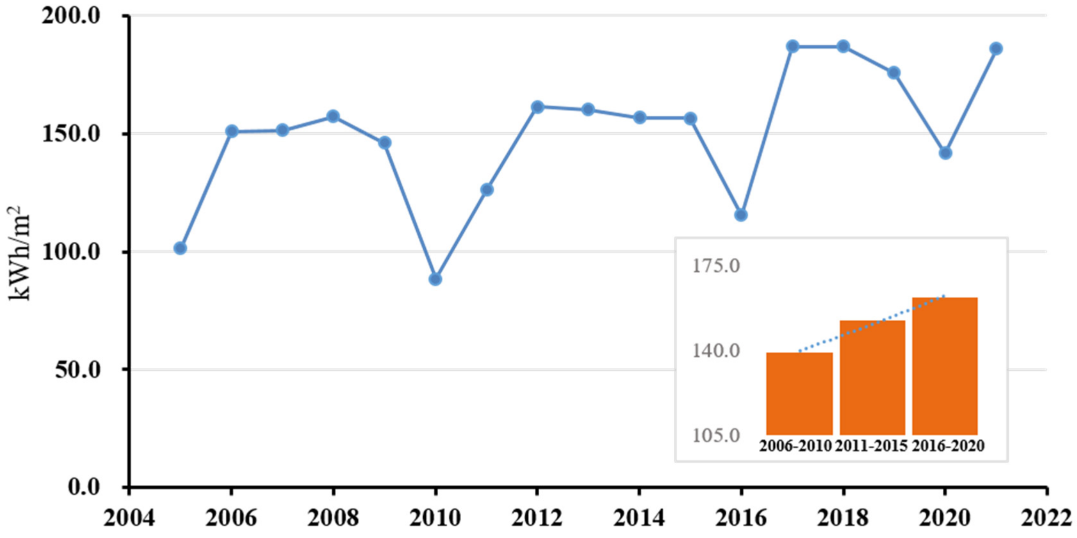

Figure S9 Energy consumption per unit area of Shenzhen Metro from 2005 to 2021.

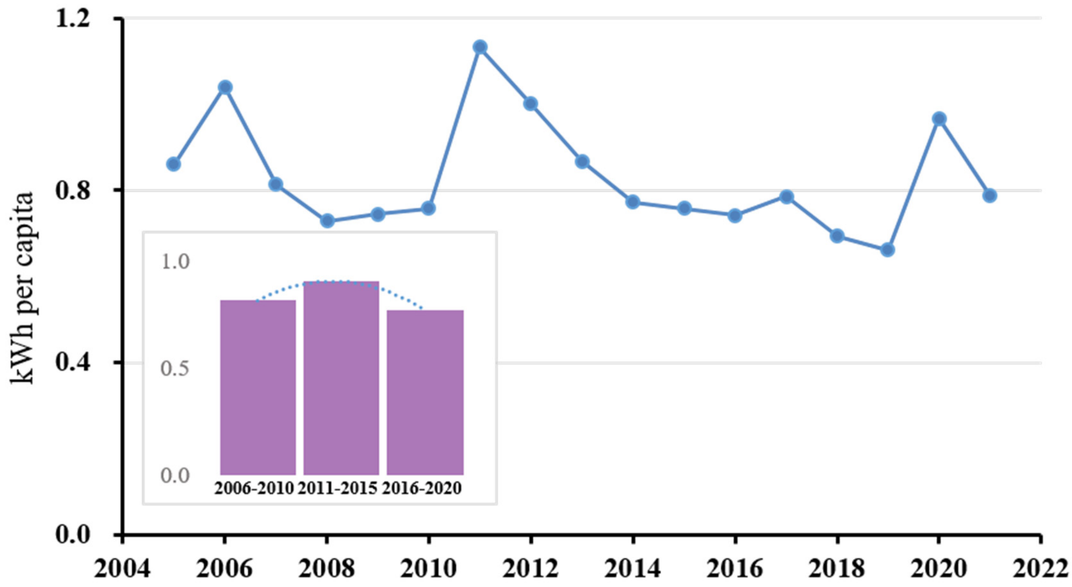

Figure S10. Energy consumption per capita of Shenzhen Metro from 2005 to 2021.
